# Raf Kinase Inhibitory Protein Protects Cells against Locostatin-Mediated Inhibition of Migration

**DOI:** 10.1371/journal.pone.0006028

**Published:** 2009-06-24

**Authors:** Anne N. Shemon, Eva M. Eves, Matthew C. Clark, Gary Heil, Alexey Granovsky, Lingchun Zeng, Akira Imamoto, Shohei Koide, Marsha Rich Rosner

**Affiliations:** 1 Ben May Department for Cancer Research, University of Chicago, Chicago, Illinois, United States of America; 2 Department of Neurobiology, Pharmacology and Physiology, University of Chicago, Chicago, Illinois, United States of America; 3 Department of Biochemistry and Molecular Biology, University of Chicago, Chicago, Illinois, United States of America; Roswell Park Cancer Institute, United States of America

## Abstract

**Background:**

Raf Kinase Inhibitory Protein (RKIP, also PEBP1), a member of the Phosphatidylethanolamine Binding Protein family, negatively regulates growth factor signaling by the Raf/MAP kinase pathway. Since an organic compound, locostatin, was reported to bind RKIP and inhibit cell migration by a Raf-dependent mechanism, we addressed the role of RKIP in locostatin function.

**Methods/Findings:**

We analyzed locostatin interaction with RKIP and examined the biological consequences of locostatin binding on RKIP function. NMR studies show that a locostatin precursor binds to the conserved phosphatidylethanolamine binding pocket of RKIP. However, drug binding to the pocket does not prevent RKIP association with its inhibitory target, Raf-1, nor affect RKIP phosphorylation by Protein Kinase C at a regulatory site. Similarly, exposure of wild type, RKIP-depleted HeLa cells or RKIP-deficient (RKIP^−/−^) mouse embryonic fibroblasts (MEFs) to locostatin has no effect on MAP kinase activation. Locostatin treatment of wild type MEFs causes inhibition of cell migration following wounding. RKIP deficiency impairs migration further, indicating that RKIP protects cells against locostatin-mediated inhibition of migration. Locostatin treatment of depleted or RKIP^−/−^ MEFs reveals cytoskeletal disruption and microtubule abnormalities in the spindle.

**Conclusions/Significance:**

These results suggest that locostatin's effects on cytoskeletal structure and migration are caused through mechanisms independent of its binding to RKIP and Raf/MAP kinase signaling. The protective effect of RKIP against drug inhibition of migration suggests a new role for RKIP in potentially sequestering toxic compounds that may have deleterious effects on cells.

## Introduction

Raf Kinase Inhibitory Protein (RKIP) is an inhibitor of key signal transduction cascades in mammalian cells that regulate growth and differentiation (reviewed in [1], [2]). RKIP binding to Raf-1 prevents MAP kinase signaling in response to growth factors [3]. Following stimulation of growth factor receptors, RKIP is phosphorylated at Ser-153 by protein kinase C (PKC), enabling RKIP dissociation and subsequent phosphorylation of Raf-1 at activating sites [4], [5]. RKIP binding to G-protein coupled receptor kinase 2, a kinase that phosphorylates and downregulates G-protein-coupled receptors, causes up-regulation of G protein-coupled receptors such as the β-adrenergic receptor [6]. RKIP has also been shown to bind NF-κB-inducing enzyme, NIK and inhibit signaling mediated by NF-κB [7]. Finally, RKIP regulates the spindle checkpoint through inhibition of the MAP kinase cascade [8]. RKIP is also missing or depleted in a number of metastatic tumors (reviewed in [1]), and has been implicated as a metastasis suppressor through regulation of one or more of these signaling cascades [9], [10].

RKIP, also termed phosphatidylethanolamine binding protein-1 (PEBP-1), is a 20–25 kDa globular protein that belongs to the PEBP family comprised of over 400 members. The crystal structures of PEPBs from bacteria to humans have revealed a remarkably conserved ligand-binding pocket [1]. In these crystal structures, phosphorylethanolamine as well as other ions are found in the ligand-binding pocket [11], and the pocket has been shown to bind hydrophobic ligands such as phospholipids [12]. While other ligands have also been reported to bind RKIP, the specific ligands that bind RKIP at physiological pH are yet to be defined.

Since RKIP regulates key physiological processes, it is of interest for therapeutic purposes to identify agents that either prevent or potentiate its ability to inhibit target proteins such as Raf-1. Recently, locostatin ((S)-(+)-4-benzyl-3-crotonyl-2-oxazolidinone) was shown to bind to RKIP and inhibit migration of Madin-Darby canine kidney (MDCK) epithelial cells [13], [14]. In this cell type, RKIP depletion also inhibited migration. Consistent with a role in Raf signaling, locostatin prevented co-immunoprecipitation of RKIP with Raf-1 *in vitro*. Overexpression of RKIP in these cells reversed the anti-migratory effect of locostatin and cells adopted a fibroblast-like phenotype [13]. More recently, locostatin was also observed to impair migration in another cell type, human breast adenocarcinoma (MCF7), as well as causing these cells to have poor cell-cell adhesive properties [14]. These results suggest that locostatin regulates cell migration via an RKIP-dependent, Raf/MAPK-regulated mechanism.

To address the role of RKIP in locostatin function, we analyzed locostatin interaction with RKIP and the biological consequences of locostatin treatment in both human and murine cell types. Our results suggest that locostatin interacts with the RKIP ligand-binding pocket but its effects on the cytoskeleton, mitotic spindle, and cell migration are independent of RKIP and the MAP kinase signal transduction cascade. Instead, RKIP appears to rescue cells from inhibition of migration by locostatin, raising the possibility that RKIP may function in part to sequester deleterious drugs.

## Results

### Locostatin precursor binds to the ligand binding pocket of RKIP

**Figure 1:**
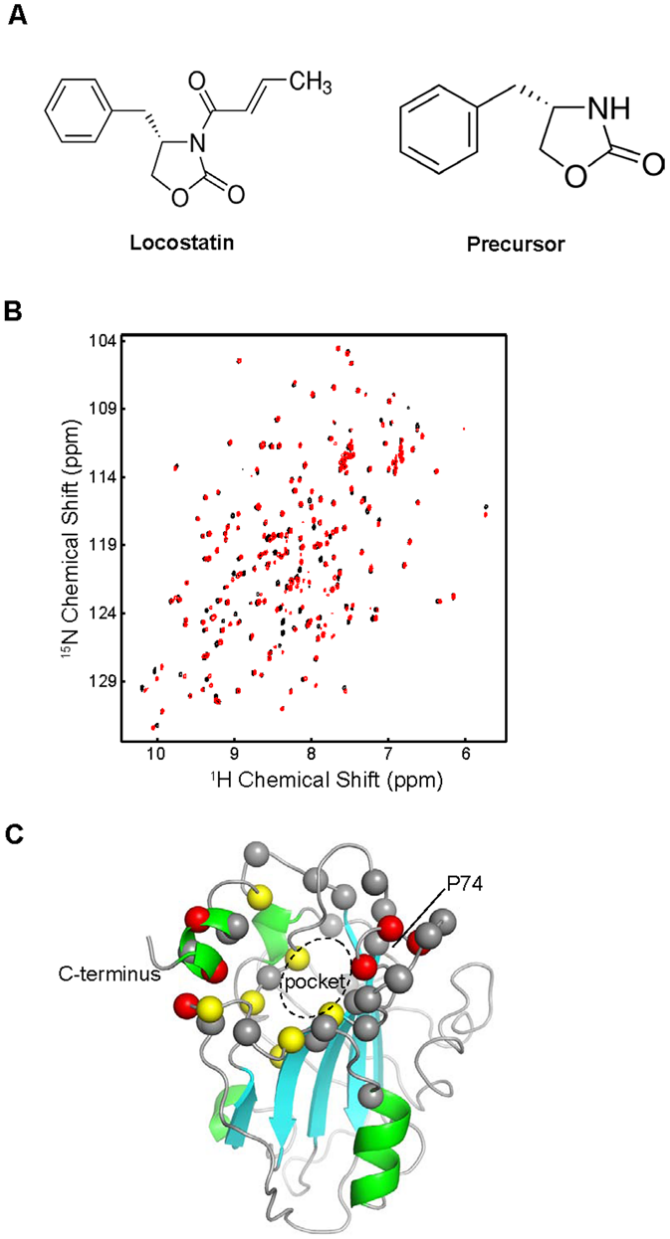
Binding of the locostatin precursor (S)-4-benzyl-2-oxazolidinone (Sigma #294640) to RKIP characterized by NMR chemical shift perturbation. (A) Chemical structure of locostatin and its precursor (S)-4-benzyl-2-oxazolidinone. (B) Comparison of ^1^H,^15^N-HSQC spectra of ^15^N-enriched RKIP in the absence-*black* and the presence-*red* of the compound (1 mM). Each cross peak (dot) represents the correlation between the directly bonded amide ^1^H and ^15^N atoms of amino acid residue. (C) The locations of amino acid residues whose HSQC peak is significantly affected by compound binding mapped on the RKIP structure. Red, yellow and gray spheres represent residues that have the weighted shift of >0.3 ppm, 0.2> ppm and >0.1 ppm, respectively.

To define the RKIP binding site for locostatin, we analyzed their interaction by NMR. We found that locostatin, a suicide inhibitor that irreversibly attached itself to RKIP, had limited solubility, and addition of locostatin to an NMR sample containing 100 µM RKIP caused RKIP precipitation, rendering NMR characterization of the RKIP-locostatin complex impossible. However, a precursor of locostatin that had the same basic structure but lacked the reactive crotonyl group, (S)-4-benzyl-2-oxazolidinone (**Fig. 1A**), was compatible with NMR studies. To detect perturbations caused by precursor binding at a single amino acid residue resolution, we obtained ^1^H,^15^N single-quantum correlation spectra (HSQC) in which each cross peak representing a directly bonded ^1^H-^15^N pair functions as an amide-specific probe in ^15^N-enriched proteins [15], [16]. Since we previously obtained the complete sequence-specific resonance assignments of backbone amides for wild-type RKIP [16], this approach enabled us to relate any perturbed HSQC cross peaks to a specific residue within RKIP. A comparison of HSQC spectra of wild-type RKIP in the presence or absence of the locostatin precursor (**Fig. 1B**) revealed that a majority of peaks affected by ligand binding were associated with the ligand-binding pocket (**Fig. 1C**). The entire rim of the pocket as well as the C-terminal alpha helix were perturbed, and the strongest perturbations seem to cluster near the C-terminal alpha helix and the conserved P74 residue on the opposite face. However, there are almost no perturbations in the region of S153, the site that, when phosphorylated, prevents RKIP binding to Raf [4], [12]. These results indicate that a locostatin-related compound indeed binds RKIP and occupies the evolutionarily conserved ligand-binding pocket of RKIP. Given the structural similarity, these results strongly suggest that locostatin also binds to the RKIP ligand-binding pocket.

### Locostatin does not alter RKIP binding to Raf-1

**Figure 2:**
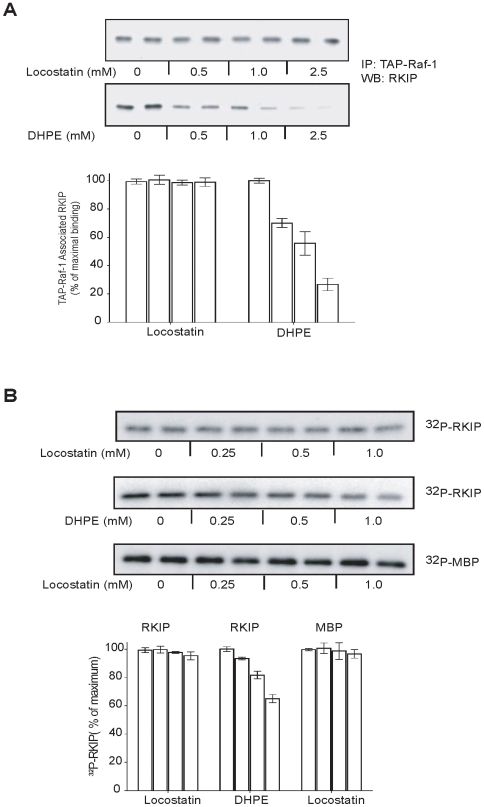
DHPE but not locostatin decreases RKIP interactions with Raf-1 and PKC. (A) RKIP association with Raf-1 in the presence or increasing concentrations of locostatin or DHPE. *Upper panels*: Representative western blots. *Lower panels*: Bar graphs representative of two independent experiments±range. (B) PKC phosphorylation of RKIP in the presence of increasing concentrations of locostatin or DHPE using RKIP or MBP as substrates. *Upper panels*: Representative autoradiogram is shown. *Lower panels*: Bar graphs representative of two independent experiments±range.

Initially we determined whether locostatin affects cellular RKIP association with Raf-1 either directly or indirectly via regulation of RKIP phosphorylation at S153. To test whether locostatin inhibits RKIP binding to Raf-1, we immunoprecipitated TAP-Raf-1 (Tandom Affinity Purification using Protein A and Flag-tagged Raf-1) that was stably expressed in H19-7 cells. The TAP-Raf-1 bound to beads was incubated with soluble, bacterially expressed RKIP in the presence of increasing concentrations of locostatin. We have used this approach previously (confirmed in **Fig. 2A**) to show that 2-dihexanoyl-sn-glycero-3-phosphoethanolamine (DHPE), a water-soluble, short-chain analog of phosphatidylethanolamine, binds to the RKIP pocket and competes with Raf-1 for association [12]. However, in contrast to DHPE, locostatin up to a concentration of 2.5 mM did not significantly inhibit RKIP binding to Raf-1 (**Fig. 2A**).

It is also possible that locostatin binding to RKIP in cells alters PKC phosphorylation of RKIP, thus indirectly affecting association of RKIP with Raf-1. To test this possibility, we determined the effect of different locostatin concentrations on RKIP phosphorylation at S153 by PKC using an *in vitro* kinase assay. Again, in contrast to DHPE [12], locostatin had no effect on RKIP phosphorylation at S153 even at higher drug concentrations (**Fig. 2B**). Taken together, these results indicate that locostatin binding to RKIP does not significantly alter RKIP binding to its inhibitory target, Raf-1.

### Locostatin does not inhibit ERK

**Figure 3:**
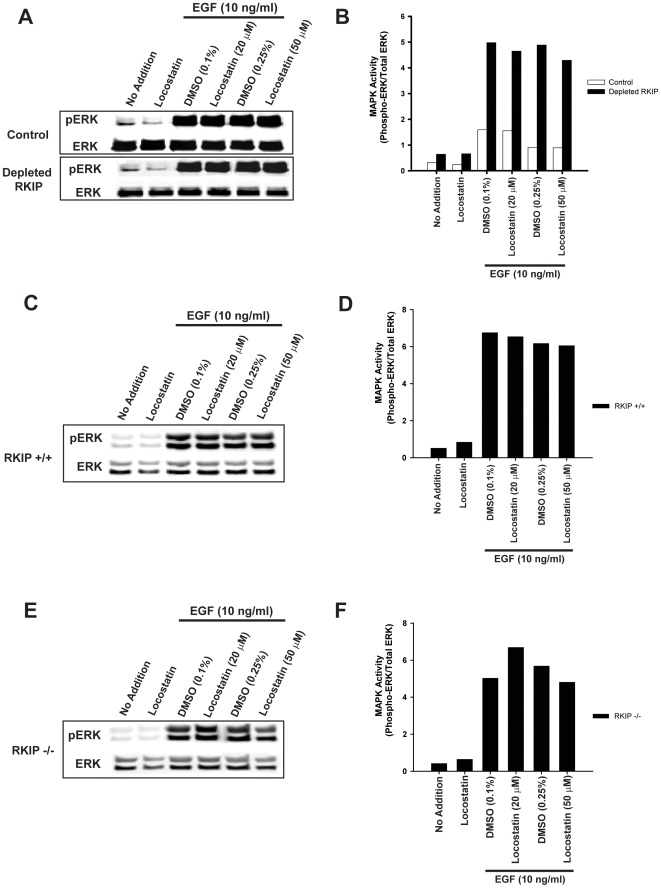
Locostatin has no effect on EGF-induced MAPK activity. (A, B) HeLa cells expressing control or depleted RKIP or (C, D) MEFs expressing wild-type RKIP (RKIP^+/+^) or (E, F) MEFs not expressing RKIP (RKIP^−/−^) were serum starved overnight and then pre-treated for 30 min with DMSO or locostatin (concentrations as indicated). EGF (10 ng/ml) was added in the final 5 minutes of incubation. Whole cell lysates (30 µg) were separated on SDS-PAGE gels (12.5%), transferred to nitrocellulose and analysed by immunoblotting with anti-phospho ERK and anti-total ERK antibodies (A, C and E). ERK phosphorylation was assessed by normalizing phospho-ERK levels to total ERK levels as depicted in bar graphs (B, D and F). The results shown are representative of three independent experiments.

We have previously shown that depletion of RKIP in HeLa cells enhances the amplitude of the ERK signal induced by epidermal growth factor (EGF) stimulation [5]. Since locostatin does not prevent RKIP association with Raf, then locostatin treatment of cells should not effect ERK signaling. Therefore, to investigate the role of locostatin in MAPK signaling, we pretreated control and RKIP-depleted HeLa cells with 20 or 50 µM locostatin for 30 min, a concentration range that was previously shown to inhibit cell migration [17]. Cells were then stimulated with EGF (10 ng/ml) in the final five minutes of incubation. As predicted, we observed a 2 to 3-fold increase in MAPK activity (measured as a ratio of phospho-ERK to total ERK) in depleted RKIP cells compared to control cells (**Fig. 3A** and **B**). Locostatin (20 or 50 µM) had no effect on basal levels of MAPK activity in these cells (Fig. 3). Similarly, locostatin failed to impair or enhance the EGF-induced MAPK activity independent of RKIP expression. An inactive analogue of locostatin that cannot bind RKIP, UIC1017 [17] also had no effect on EGF-induced MAPK activity in these cells (data not shown).

To further confirm that any locostatin effects on cells are independent of RKIP or MAPK activation status, we also tested the effect of locostatin on mouse embryonic fibroblasts (MEFs) isolated from either wild type (RKIP^+/+^) or homozygous (RKIP^−/−^) mice. As observed with HeLa cells, neither 20 µM nor 50 µM locostatin had any effect on EGF-induced MAPK activity in MEFs that express or are deficient in RKIP (**Fig. 3C–F**). These results indicate that locostatin binding to the RKIP ligand pocket does not perturb RKIP/Raf-1 association nor alter MAPK activity.

### RKIP protects against locostatin inhibition of cellular migration after wound healing in MEFs

**Figure 4:**
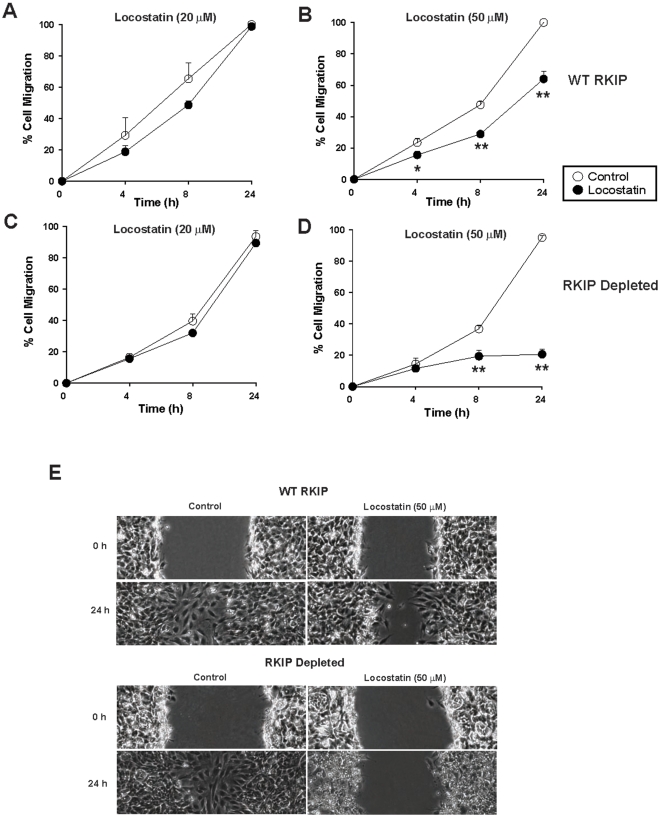
Locostatin impairs wound healing in wild-type and depleted RKIP MEFs. MEFs expressing wild-type (A, B) or depleted (C, D) RKIP were seeded at 0.5×10^6^/ml and grown for 24 h before treatment with either DMSO (control) or locostatin at 20 (A) and (C) or 50 µM (B) and (D). (E) A standardized scratch (wound) was applied to monolayers and digital images were taken at time points indicated. Cell migration was determined as described in materials and methods. Data represents the mean±SEM of eight wounds from two independent experiments. ^*^p<0.05, ^**^p<0.01 indicates the significance of the change relative to the corresponding sample in the absence of locostatin.

**Figure 5:**
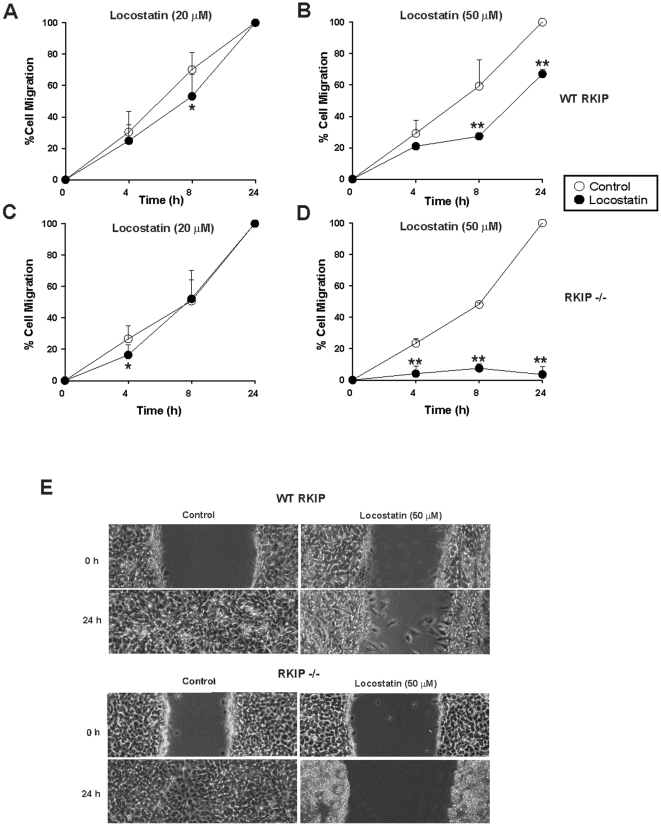
Locostatin impairs wound healing independent of RKIP. MEFs expressing wild-type (A, B) or deficient (C, D) RKIP were seeded at 0.5×10^6^/ml and grown for 24 h before treatment with either DMSO (control) or 20 (A) and (C) or 50 µM (B) and (D) locostatin. (E) A standardized scratch (wound) was applied to monolayers and analyzed as in materials and methods. Data represents the mean±SEM of three independent experiments (n = 4 wounds/sample). ^*^p<0.05, ^**^p<0.01 indicates the significance of the change relative to the corresponding sample in the absence of locostatin.

Previous reports using MDCK and MCF-7 cells have shown that 50 µM locostatin inhibits the rate of cell migration of cultured cells following wounding [14]. To determine whether locostatin inhibits migration in another cell type, we established a scratch wound assay using RKIP^+/+^ MEFs (**Fig. 4**). MEFs were treated with DMSO (vehicle control) or two different doses of locostatin and the wounds were visualized using digital images at 0, 4, 8 and 24 h time points. In the presence of vehicle (0.25% DMSO), the cells healed within 24 h (**Fig. 4A**) with no signs of cell toxicity as determined by trypan blue exclusion (data not shown). Similarly, 20 µM locostatin had no effect on migration (**Fig. 4A**), as opposed to 50 µM locostatin which impaired wound healing at all time points measured (**Fig. 4B**). Locostatin (50 µM) when compared to vehicle (0.25% DMSO) treatment (24 h time point) reduced migration by 40±5% (n = 8 separate wounds) in RKIP^+/+^ MEFs (**Fig. 4B**). Surprisingly, depletion of RKIP (depletion described here refers to short-hairpin RNA interference with RKIP in MEFs) further reduced migration to 77±3% at this concentration of locostatin (**Fig. 4D**). To confirm that remaining RKIP in the depleted MEFs was not responsible for the observed effects, we also tested RKIP deficient MEFs (RKIP^−/−^). Total loss of RKIP almost completely inhibited migration in the presence of 50 µM locostatin, 96±5% at 24 h when compared to RKIP^+/+^; p<0.01; compare **Fig. 5B** and **D**). Furthermore, the rate of migration in the presence of vehicle was not significantly affected by the presence or absence of RKIP. Therefore, these results indicate that 50 µM locostatin is sufficient to inhibit migration, and RKIP confers significant protection to cells within this concentration range.

### Locostatin impairs cytoskeletal organization, disrupts the mitotic spindle and promotes nuclear fragmentation

**Figure 6:**
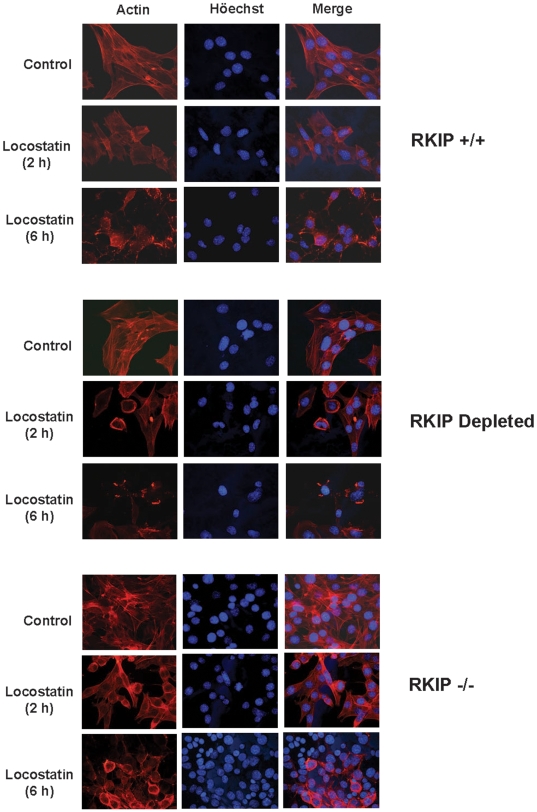
Locostatin impairs cytoskeletal organization independent of RKIP expression. MEFs expressing wild-type, depleted or no RKIP were plated at 2×10^4^ cells/coverslip and grown for 48 h before treatment with either DMSO (Control) or locostatin (50 µM) for either 2 or 6 h. Cells were fixed and permeabilized and stained with phalloidin for actin-*red*. DNA/chromatin was stained with Höechst 33342-*blue*. Digital photomicrographs were captured using Openlab Darkroom (Improvision) and a Retiga 1300 color digital camera (Q Imaging). Images from MEFs expressing wild-type RKIP were similar to the RKIP^+/+^ MEFs which express lentiviral vector control. Intensity of images were adjusted to display qualitative rather than quantitative comparisons so that the intensity of staining in each panel cannot be directly compared.

Since locostatin has been previously reported to inhibit cell migration, its physiological effects are likely mediated, at least in part, via cytoskeletal interactions. To investigate the cytoskeleton we pretreated MEFs of varying genotypes with locostatin (50 µM) and stained them with phalloidin to locate actin filaments within the cell. Previous studies [17] reported that locostatin did not affect the formation of new filamentous actin bundles in MDCK cells post wounding. By contrast, we observed a time dependent induction of cytoskeletal reorganization by locostatin in MEFs (**Fig. 6**). In vehicle control MEFs (RKIP^+/+^, depleted and RKIP^−/−^), cells stained positive for filamentous F-actin with an even distribution of F-actin around the nucleus as well as along stress fibers within the cell (**Fig. 6**). In the presence of locostatin (50 µM) we observed a slight decrease in actin staining after 2 h of treatment, and by 6 h actin was predominantly localized to the perinuclear region of the cell (**Fig. 6**). Similarly, RKIP depleted cells displayed a locostatin effect; however, by 6 h it was evident that locostatin caused the collapse of the cytoskeleton as evidenced by near absent actin staining throughout the cell (**Fig. 6**). Likewise, locostatin caused reduced actin staining in RKIP deficient (RKIP^−/−^) MEFs (**Fig. 6**, lower panel). As observed with migration, the effect of locostatin was more pronounced in RKIP^−/−^ MEFs compared to RKIP^+/+^. By 6 h, treatment with locostatin in all genotypes exhibited disrupted actin cytoskeleton as detected by phalloidin. However, following 2 h treatment with locostatin the RKIP^+/+^ cells looked similar to vehicle control cells, whereas the RKIP depeleted or RKIP^−/−^ cells already displayed defects in the actin cytoskeleton. Thus, RKIP appears to transiently protect cells from locostatin mediated disruption of the actin cytoskeleton.

**Figure 7:**
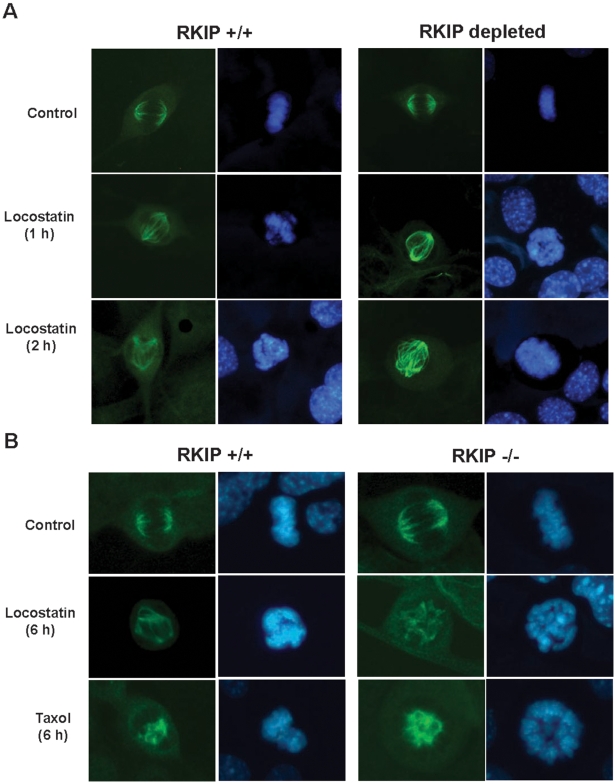
Locostatin disrupts the mitotic spindle and chromosome organization. MEFs expressing (A) wild-type (RKIP^+/+^) or lacking RKIP were plated at 2×10^4^ cells/coverslip and grown for 48 h before treatment with either DMSO (Control) or locostatin (20 µM) for either 1 or 2 h. (B) Wild-type (RKIP^+/+^) or deficient (RKIP^−/−^) RKIP were plated as described in (A) and treated with either DMSO (Control), locostatin (20 µM) or paclitaxel (10 µM) for 6 h. Cells were fixed and permeabilized and stained with anti-α-tubulin antibody-*green*. DNA/chromatin was stained with Höechst 33342-*blue*. Digital photomicrographs were captured using Openlab Darkroom (Improvision) and a Retiga 1300 color digital camera (Q Imaging). Intensity of images were adjusted to display qualitative rather than quantitative comparisons so that the intensity of staining in each panel cannot be directly compared.

The mitotic spindle is composed of microtubules that are highly sensitive to both polymerizing and depolymerizing agents, and therefore could serve as a sensor for microtubule-dependent cytoskeletal changes. To test this possibility, we determined whether locostatin affects mitotic spindle structure. RKIP^+/+^ MEFs or RKIP-depleted MEFs were either untreated or treated with locostatin (20 µM) for the indicated time (**Fig. 7A**) and cells were fixed and stained with anti-α tubulin antibody. As shown in **Fig. 7A** locostatin caused a time dependent loss of spindle fiber tension in RKIP^+/+^ and RKIP-depleted cells. Both RKIP^+/+^ and RKIP^−/−^ MEFs were treated for 6 h with locostatin (20 µM) which showed defects in mitotic spindle formation and chromosome organization (**Fig. 7B**). Although abnormal spindles were observed after 2 h treatment with 20 µM locostatin in all cell types, there were fewer abnormal spindles following 1 h treatment in RKIP expressing cells (supplemental **Fig. S1**). We also observed at this dose of locostatin a disruption in chromatin condensation in a manner reminiscent of Taxol, a drug that causes loss of spindle fiber tension (**Fig. 7B**). This effect was independent of RKIP expression, indicating that locostatin targets cytoskeletal elements through a mechanism that does not involve RKIP binding or inhibition of RKIP function.

## Discussion

In this study, we have demonstrated that locostatin inhibits migration in MEFs independent of RKIP expression and Raf/MAPK signaling. The locostatin precursor binds to RKIP's ligand-binding pocket but has no effect on Ser-153, the known site for PKC phosphorylation of RKIP that leads to dissociation of RKIP from Raf-1 [4]. Similarly, increasing concentrations of locostatin and its precursor had very little effect on RKIP phosphorylation by PKC or RKIP/Raf-1 association. Finally, locostatin in RKIP-depleted and RKIP-deficient cell lines (HeLa and MEFs) had no effect on the EGF-induced activity of MAPK, a downstream signaling target of Raf. Furthermore, our data shows that locostatin is not specific to RKIP and has off target effects. In particular, locostatin reorganizes the actin cytoskeleton, disrupts the mitotic spindle and inhibits cell migration via a mechanism that is independent of the MAPK pathway. The protective effect of RKIP toward locostatin disruption of the cytoskeleton and inhibition of migration indicates that RKIP can influence locostatin action but not necessarily as a mediator. Our results raise the possibility that RKIP may function to trap locostatin, sequestering the toxic drug within the cell.

Our results add a different perspective to our understanding of locostatin interaction with RKIP. Previous studies have shown that locostatin prevents RKIP from co-precipitating with Raf-1. However, it is possible that the chemically reactive nature of locostatin may have caused other interactions that sterically hinder Raf access to RKIP. The deleterious effects of locostatin on cytoskeletal structures and cell migration even in cells lacking RKIP suggest that locostatin has other targets that may not have been previously identified. One puzzling observation is that locostatin inhibits spindle organization and cell migration at 20–50 µM but the precursor binds to RKIP at millimolar concentrations. The reduced apparent affinity of the precursor may be attributable to the different modes of interaction between the two compounds. Locostatin acts as a suicide inhibitor that covalently attaches itself to RKIP, while the precursor lacks a chemically reactive group and binds RKIP in a reversible manner [13]. An alternative explanation for these differences may be the size of locostatin compared to its precursor, which may also cause differences in their effects on cell migration or cytoskeleton, unfortunaletly solubility constraints for the precursor limited our study in an intact cell system. The effect of locostatin is highly concentration-dependent; just a two-fold decrease in dose caused loss of inhibition. Thus, a reduction of the effective concentration of locostatin in cells could significantly diminish its efficacy. RKIP may be present in the cell at sufficient concentrations to promote RKIP-locostatin interaction and sequester the toxic drug. Alternatively, it is also possible that RKIP plays a discrete role in cytoskeletal organization and migration that counteracts the effects of locostatin.

Cell migration is a key step underlying cellular development as well as other physiological and disease processes but whether RKIP plays a role in regulating this process is still unresolved. In some studies, RKIP overexpression inhibits migration and invasion [18], [19]. In other reports, RKIP promotes cell migration by downregulating E cadherin and upregulating β1 integrin [13], [14]. In the present study using MEFs, the presence or absence of RKIP has no effect on the rate of wound healing. The inhibitory action of locostatin on wound healing in the MEFs appears to be associated with changes in the cytoskeleton. We stained cells of varying RKIP genotypes for actin with phalloidin and observed the shortening of stress fibers within the cytoplasm and a concentration of actin around the nucleus. These were exacerbated in MEFs with depleted or absent RKIP and these cells had an increased amount of nuclear bodies with very little actin association compared to their wild-type counterparts. Similarly, the inhibitory effect of locostatin on migration and cytoskeleton was more pronounced when cells were grown to a lower density as opposed to the confluent monolayers described by [17], and we also observed interbatch variations of locostatin potency. Collectively, these discrepancies may reflect differences in cell type and/or assays and will require further analysis of multiple tissues from RKIP knockout mice.

At the doses of locostatin used in this study, we have shown that RKIP may not be the only locostatin target regulating cell migration. Previously, [^3^H]locostatin was shown in an association assay to bind to four proteins only, one of which was RKIP [13]. The other proteins were omega 1-1, prolyl endopeptidase and aldehyde dehydrogenase (ALDH1A1). However, they reported that ALDH1A1, which belongs to the ALDH family of enzymes and is known to clear the cell of toxic reactive oxygen species byproducts predominantly in the liver [20], bound to locostatin but was not involved in cell migration. Interestingly, ALDH1A1 was recently shown to mediate cell invasion in pancreatic cancer cells [21] suggesting that it does play a role in cytoskeletal rearrangement. Therefore, these results raise the question whether ALDH1A1 might function to bind locostatin as a toxic byproduct for its removal from the cell. If this were true, ALDH1A1 function would complement a role for RKIP in binding to locostatin to mop up the drug and subsequently dampen its anti-migratory effect on the cell.

To our knowledge this report provides the first evidence that locostatin can alter both microtubule (tubulin) and actin-based cytoskeletal elements. We observed that locostatin interferes with the cytoskeleton of MEFs since it caused the collapse of the actin cytoskeleton within the cell and localized actin to perinuclear regions. Locostatin also disrupted mitotic spindle formation in MEFs similar to the effects of Taxol. These results suggest that locostatin plays a role in altering the cytoskeleton via a yet to be defined mechanism warranting future investigation.

The present study of locostatin binding to RKIP also provides insight into the role of the pocket in Raf-1 interactions. The precursor bound directly to the pocket as shown by the perturbation of key residues in the RKIP ligand binding pocket. Although locostatin's binding to RKIP could not be examined directly by NMR due to its aggregation at high concentrations, it is likely that locostatin binds to the same RKIP pocket residues. The data show precursor binding to the RKIP pocket is not sufficient to interfere with Raf binding, consistent with the observed MAPK signaling in locostatin-treated cells. This result is in contrast to the lipid DHPE that not only binds the pocket but also inhibits RKIP-Raf association. Comparison of these different outcomes suggests that other residues and/or steric features of RKIP as well as size of the ligand are critical for Raf-1 binding [12].

RKIP is a negative regulator of major signaling cascades including MAPK; therefore, the identification of drugs that bind to RKIP and influence its inhibitory function could represent an important therapeutic strategy. Although locostatin is the first described drug to bind RKIP, it is likely that other chemicals also interact with RKIP via the ligand binding pocket. The studies here suggest that it may be possible to find therapeutic agents that either potentiate or suppress RKIP association with kinases and its inhibitory targets. Further studies to characterize the nature of the interactions will be needed to identify the most effective RKIP-modulating drugs. This work also suggests that RKIP might serve as a “reservoir” for hydrophobic chemicals circulating in the blood.

## Materials and Methods

### Reagents and antibodies

(S)-(+)-4-benzyl-3-crotonyl-2-oxazolidinone (locostatin) and its precursor, (S)-4-benzyl-2-oxazolidinone (**Fig. 1A**) used in NMR studies were from Aldrich (St. Louis, MO). Protease inhibitor cocktails and locostatin unless otherwise indicated were from Calbiochem. All cell culture media and supplements were from GIBCO (Invitrogen, Carlsbad, CA). ^15^N-ammonium chloride was from Cambridge Isotope Laboratories (Andover, MA). Anti-phospho and -total ERK antibodies were from Cell Signaling Technology (Beverly, MA). Anti-rabbit and –mouse IR dye secondary antibodies as well as Odyssey blocking buffer used for Western blotting were purchased from LI-COR Biosciences (Lincoln, NE). Anti-α tubulin (B-7) mouse antibody was purchased from Santa Cruz Technologies (Santa Cruz, CA). Anti-RKIP antibody was developed as described [4]. FITC-conjugated donkey anti-mouse secondary antibody was from Jackson Immunolabs (West Grove, PA). Höechst 33342 nucleic acid stain and Alexafluor 594-tagged phalloidin were from Molecular Probes (Eugene, OR) and mounting media was Vectashield Hardset from Vector Labs (Burlingame, CA). IgG sepharose beads were from Amersham Biosciences (GE Healthcare, Piscataway, NJ). Biotinylated thrombin (50 U) was from Novagen (Madison, WI).

### Cell lines and cell culture

HeLa cells stably expressing short hairpin RNA vectors for depleting human RKIP or rat RKIP control were maintained at 37°C/5% CO_2_ in Dulbecco's modified Eagle's medium (DMEM) supplemented with 10% fetal bovine serum, 50 U/ml penicillin and 50 µg/ml streptomycin under puromycin (2 µg/ml) selection as previously described [5]. Mouse embryonic fibroblasts (MEFs) expressing varying genotypes of RKIP (wild-type RKIP^+/+^ or homozygous RKIP^−/−^) were isolated from knockout RKIP mice that were generated using embryonic stem cells established by the Sanger Center using a gene targeting approach similar to that previously described [22]. MEFs were isolated from 12.5-day-old embryos that were derived from intercross of RKIP^+/−^ mice. The generation and characterization of RKIP knockout mice and MEFs will be reported elsewhere. MEFs were immortalized with a temperature sensitive SV40 large T-antigen. RKIP^+/+^ MEFs were depleted by transduction with shRNA vectors using a pLKO.1 lentiviral vector as previously described [10]. These cells were maintained in culture medium (as described for HeLa cells under 200 µg/ml geneticin and/or 2.5 µg/ml puromycin selection at 33°C/5% CO_2_.

### NMR analysis

^15^N-Enriched RKIP was prepared by expressing the GST-RKIP fusion protein in M9 minimal media supplemented with ^15^N-ammonium chloride. After purification, RKIP was excised from the fusion protein using thrombin and concentrated. The NMR sample contained 100 µM ^15^N-RKIP in 50 mM Tris HCl buffer (pH 7.4) containing 100 mM NaCl prepared in 93% H_2_O and 7% D_2_O. ^1^H,^15^N-HSQC spectra were acquired at 30°C on a Varian Inova 600 spectrometer equipped with a cryogenic probe. NMR data were processed using the NMRPipe suite [23] and analyzed using the NMRView program [24]. Sequence-specific resonance assignments for RKIP have been published (BMRB accession code 6783) [16]. The weighted shift, ((^1^H shift)^2^+0.17(^15^N shift)^2^)^1/2^, was calculated following the method of Farmer *et al*. [25].

### In vitro Raf and PKC kinase assay

The pRav-Flag-Raf-1 plasmid (TAP-Raf-1; Tandem Affinity Purification using Protein A and Flag tagged-Raf-1) was constructed as previously described [12] and stably expressed in H19-7 cells. Briefly, TAP-Raf-1 was immunoprecipitated from H19-7 cells that were serum starved overnight in DMEM media with no supplements, and TAP-Raf was isolated on IgG sepharose beads. Cells were lysed in TAP-lysis buffer (10 mM HEPES pH 7.4, 3 mM MgCl_2_, 10 mM KCl, 5% Glycerol, 0.1% NP40) and cleared by centrifugation. Cell lysates were combined with pre-equilibrated TAP-lysis buffer IgG sepharose beads and incubated for 1 h at 4°C. Beads were washed with TAP-lysis buffer and equilibrated with binding buffer (10 mM HEPES pH 7.4, 3 mM MgCl_2_, 10 mM KCl, 150 mM NaCl). Beads were aliquoted and combined with RKIP (5 µg) and increasing concentrations of locostatin or 1,2-dihexanoyl-sn-glycero-3-phosphoethanolamine (DHPE).and incubated for 30 min at 4°C. Complexes were washed three times with binding buffer with corresponding concentrations of locostatin or DHPE and boiled in sample buffer (2×). Lysates were separated on SDS-PAGE (12%), transferred to nitrocellulose and immunoblotted with anti-RKIP antibody.

### RKIP phosphorylation

*In vitro* phosphorylation of RKIP by PKCα was as previously described [12]. Briefly, RKIP (5 µg) was combined with increasing concentrations of locostatin or DHPE and the reaction was initiated with 100 mM ATP containing 5 µCi of [γ-^32^P]ATP at 30°C for 10 min. The reaction was terminated using sample buffer (6×) and boiling at 100°C for 5 min. Lysates were separated on SDS-PAGE (12%), transferred to nitrocellulose and visualized by exposing to film. Results were quantified using phospho-imager screens (Molecular Dynamics) and analyzed with ImageQuant software. Membranes were subsequently immunoblotted with anti-RKIP antibody [4].

### MAPK activation assay

HeLa cells or MEFs (RKIP^+/+^ or RKIP^−/−^) were seeded in 6-well plates at 0.5×10^6^/ml and allowed to adhere overnight at 37°C or 33°C respectively [5], [10]. Cells were serum starved overnight in DMEM media with no supplements. The next day cells were treated with DMSO (0.1 or 0.25%) or locostatin (20 or 50 µM) for 25 min followed by EGF (10 ng/ml) for 5 min at 37°C or 33°C respectively. The reaction was terminated by placing plates on ice and washing with cold PBS (containing protease inhibitors). Cells were homogenized by cell scraping in RIPA lysis buffer supplemented with sodium orthovanadate (0.1 mM), sodium fluoride (0.5 mM) and protease inhibitor mixture tablet. Cell lysates were sheared five times in a 1 ml tuberculin syringe attached with a 21 G×19 mm needle, left on ice for 1 h and then spun at 16, 000×*g* for 10 min at 4°C. Cell lysates (30 µg) were resolved by SDS-PAGE (12.5%), transferred to nitrocellulose and analysed by Western blotting with anti-phospho ERK (Cell Signaling Technology, Inc) or anti-total ERK (Cell Signaling Technology, Inc) primary antibodies. Membranes were probed with anti-rabbit and –mouse IR dye secondary antibodies (LI-COR Biosciences). The amount of MAPK activity determined for each sample was normalized to total ERK in each sample. Digital analysis of immunoreactivity was done using LI-COR Biosciences Infrared Imaging System which is quantitative and independent of exposure time. Odyssey software (version 2.1; Lincoln, NE) was used for analysis of the immunoblots.

### Scratch wound assay

MEFs of varying genotypes (as indicated) were seeded at 0.5×10^6^/ml in 6-well plates under selection as described above and allowed to adhere for 24 h at 33°C. On the day of the assay wells were rinsed once with warm PBS and then kept in supplemented DMEM media in the absence or presence of DMSO (0.1 or 0.25%) or locostatin (20 or 50 µM) at 33°C for 24 h. Wounding was performed using a pipette tip and by drawing four wounds of standard size across a confluent monolayer of cells. Prior to incubation at 33°C/5% CO_2_, digital images were taken at time zero followed by four, eight and 24 h. Images were taken on a phase-contrast microscope (Leica DMIRB, epifluorescence) at ×5 magnification. Wound area was determined as a percentage of the wound size at time zero and using the area of the wound as measured by Image J software (NIH). Initial wound area was standardized for all conditions tested and then changes in area over time were expressed as a percentage of the initial time zero wound.

### Actin localization and mitotic spindle microscopy

MEFs of varying genotypes (as indicated) were plated at 2×10^4^ cells per 15 mm coverslip and grown for 48 h. Cells were treated with DMSO only (Control), or locostatin (20 or 50 µM), or paclitaxel (10 µM) for 1 to 6 h (as indicated), followed by fixation with 4% paraformaldehyde (in 0.1 M Na-phosphate, pH 7.4) for 10 mins at room temperature (RT), permeabilized with 0.1% TritonX-100 in PBS for 2 mins at RT, and blocked with 3% BSA in PBS overnight at 4°C. Alpha-tubulin was detected using a mouse antibody at 1∶50 dilution and actin was detected using Alexafluor 594-tagged phalloidin (4 U/ml) in 1% BSA in PBS for 2 h at RT. The cells were washed 3 times with 1% BSA in PBS, and the tubulin antibody detected with FITC-conjugated donkey anti-mouse antibody at 1∶100 in 1% BSA in PBS for 1 h at RT. The cells were then washed 3 times with PBS. The second PBS wash contained 1 µg/ml Höechst 33342 to visualize DNA/chromatin. The coverslips were mounted on slides using Vectashield Hardset. Digital photomicrographs were captured using Openlab Darkroom (Improvision) and a Retiga 1300 color digital camera (Q Imaging).

### Statistical analysis

Differences between locostatin treated or untreated cells were compared using a two-tailed unpaired Student's t-test.

## Supporting Information

Figure S1 Locostatin disrupts the mitotic spindle and chromosome organization. MEFs expressing (A) wild-type (RKIP+/+) or lacking RKIP (RKIP depleted or RKIP−/−) were plated at 2×104 cells/coverslip and grown for 48 h before treatment with either DMSO (Control) or locostatin (20 µM) for either 1 or 2 h. Data represents the mean±S.E. percent abnormal mitotic spindles (n = 3), cells counted ranged from 33–119 for the various cell lines.
(0.04 MB TIF)
