# Supplementary figures and images for: Raf Kinase Inhibitory Protein Protects Cells against Locostatin-Mediated Inhibition of Migration
Source: PLoS One. 2009 Jun 24;4(6):e6028. doi: 10.1371/journal.pone.0006028 (PMC2696091; doi:10.1371/journal.pone.0006028)

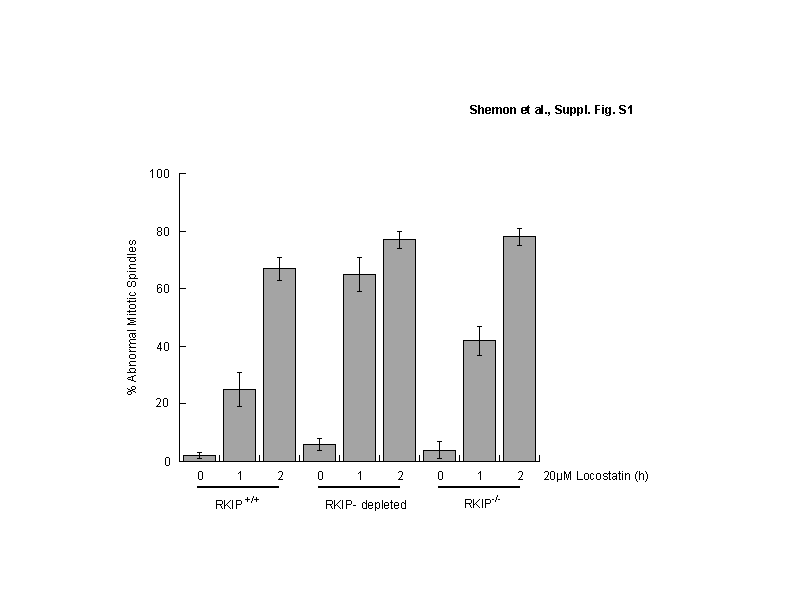

Supplement: Figure S1 — Locostatin disrupts the mitotic spindle and chromosome organization. MEFs expressing (A) wild-type (RKIP+/+) or lacking RKIP (RKIP depleted or RKIP−/−) were plated at 2×10^4^ cells/coverslip and grown for 48 h before treatment with either DMSO (Control) or locostatin (20 µM) for either 1 or 2 h. Data represents the mean±S.E. percent abnormal mitotic spindles (n = 3), cells counted ranged from 33–119 for the various cell lines. (0.04 MB TIF) [file pone.0006028.s001.tif]
